# Are There Any Pleiotropic Benefits of Vitamin D in Patients With Diabetic Kidney Disease? A Systematic Review of Randomized Controlled Trials

**DOI:** 10.1177/20543581231212039

**Published:** 2023-11-28

**Authors:** Jaya K. Sharma, Sono Khan, Tristin Wilson, Nathan Pilkey, Sanjana Kapuria, Angélique Roy, Michael A. Adams, Rachel M. Holden

**Affiliations:** 1 Department of Biomedical and Molecular Sciences, Queen’s University, Kingston, ON, Canada; 2 Bracken Health Sciences Library, Queen’s University, Kingston, ON, Canada; 3 Department of Medicine, Queen’s University, Kingston, ON, Canada

**Keywords:** 25-hydroxyvitamin D, diabetic kidney disease, diabetes

## Abstract

**Background:**

Type 2 diabetes (T2D) and kidney disease are risk factors for vitamin D deficiency. Native forms of vitamin D have a lower risk of hypercalcemia than calcitriol, the active hormone. The enzyme responsible for activating native vitamin D is now known to be expressed throughout the body; therefore, native vitamin D may have clinically relevant effects in many body systems.

**Objective:**

The objective of this systematic review was to examine the effect of native vitamin D supplementation on clinical outcomes and surrogate laboratory measures in patients with T2D and diabetic kidney disease (DKD).

**Design:**

Systematic review.

**Setting:**

Randomized controlled trials (RCTs) conducted in any country.

**Patients:**

Adults with T2D and DKD receiving supplementation with any form of native vitamin D (eg, ergocalciferol, cholecalciferol, calcifediol).

**Measurements:**

Clinical outcomes and surrogate clinical and laboratory measures reported in each of the trials were included in this review.

**Methods:**

The following databases were searched from inception to January 31, 2023: Embase, MEDLINE, Cochrane CENTRAL, Web of Science, ProQuest Dissertations and Theses, and medRxiv. Only RCTs examining supplementation with a native vitamin D form with a control or placebo comparison group were included. We excluded studies reporting only vitamin D status or mineral metabolism parameters, without any other outcomes of clinical relevance or surrogate laboratory measures. Study quality was evaluated using the Cochrane risk-of-bias tool (RoB2). Results were synthesized in summary tables for each type of outcome with the *P* values from the original studies displayed.

**Results:**

Nine publications were included, corresponding to 5 separate RCTs (377 participants total). Mean age ranged from 40 to 63. All trials administered vitamin D_3_. Intervention groups experienced improvements in vitamin D status and a reduction in proteinuria in 4 of the 5 included RCTs. There was a decrease in low-density lipoprotein and total cholesterol in the 2 trials in which they were measured. Improvements in bone mass, flow-mediated dilation, and inflammation were also reported, but each was only measured in 1 RCT. Effects on glucose metabolism, high-density lipoprotein, triglycerides, blood pressure, oxidative stress, and kidney function were mixed. No serious adverse effects were reported.

**Limitations:**

Limitations include the small number of RCTs and lack of information on the use of drugs that affect measured outcomes (eg, proteinuria-lowering renin-angiotensin-aldosterone system inhibitors and lipid-lowering medication) in most studies. Our study is also limited by the absence of a prestudy protocol and registration.

**Conclusions:**

Native vitamin D is a safe treatment that improves vitamin D status in patients with DKD. Vitamin D may modify proteinuria and lipid metabolism in DKD, but further well-designed trials that include well-established treatments are necessary. Overall, there is limited evidence for beneficial pleiotropic effects of vitamin D in patients with DKD.

## Abrégé

**Contexte:**

Le diabète de type 2 (DT2) et l’insuffisance rénale sont des facteurs de risque pour une carence en vitamine D. Les formes natives de la vitamine D représentent un risque plus faible d’hypercalcémie que le calcitriol, la forme active sur le plan hormonal de la vitamine D. On sait maintenant que l’enzyme responsable de l’activation de la vitamine D peut être exprimée dans tout le corps et donc, que la vitamine D native peut avoir des effets cliniquement significatifs dans de nombreux systèmes de l’organisme.

**Objectif:**

Examiner l’effet d’une supplémentation en vitamine D native sur les résultats cliniques et les mesures de laboratoire de substitution de patients atteints de DT2 et de maladie rénale diabétique (MRD).

**Conception:**

Revue systématique

**Sources:**

Les essais contrôlés randomisés (ECR) pertinents, sans égard au pays où ils ont été menés.

**Sujets:**

Des adultes atteints de DT2 et de MRD recevant une supplémentation de toute forme de vitamine D native (ergocalciférol, cholécalciférol, calcifédiol).

**Mesures:**

Les mesures biologiques et cliniques de substitution ainsi que les résultats cliniques rapportés dans chacun des essais inclus.

**Méthodologie:**

Une recherche des articles pertinents a été effectuée dans les bases de données Embase, MEDLINE, Cochrane CENTRAL, Web of Science, ProQuest Dissertations and Theses et medRxiv depuis leur création jusqu’au 31 janvier 2023. Seuls les ECR examinant la supplémentation avec une forme native de vitamine D contre un groupe témoin ou un placebo ont été inclus. Nous avons exclu les études ne rapportant que le statut en vitamine D ou les paramètres du métabolisme minéral, sans aucun autre résultat significatif sur le plan clinique ou mesure de laboratoire de substitution. La qualité des études a été évaluée à l’aide de l’outil Cochrane sur le risque de biais (RoB2). Les résultats ont été résumés dans des tableaux récapitulatifs pour chaque type de résultat avec les valeurs de p tirées des essais originaux.

**Résultats:**

Neuf publications ont été incluses, lesquelles portaient sur cinq ECR distincts (377 participants au total). L’âge moyen des sujets variait de 40 à 63 ans. De la vitamine D_3_ avait été administrée dans tous les essais. Dans quatre des cinq ECR inclus, le groupe d’intervention avait connu une amélioration du statut en vitamine D et une réduction de la protéinurie. Une diminution des LDL et du cholestérol total avait été observée dans les deux essais où ces paramètres avaient été mesurés. Des améliorations de la masse osseuse, de la dilatation médiée par le débit et de l’inflammation avaient également été rapportées, mais chacun de ces paramètres n’avait été mesuré que dans un seul ECR. Lorsque rapportés, les effets sur le métabolisme du glucose, les HDL, les triglycérides, la pression artérielle, le stress oxydatif et la fonction rénale étaient mitigés. Aucun effet indésirable grave à la supplémentation n’a été signalé.

**Limites:**

Les résultats sont limités par le faible nombre d’ECR inclus et par le manque d’information dans la plupart des études sur l’utilisation de médicaments qui affectent les résultats mesurés (par exemple, les inhibiteurs du SRAA abaissant la protéinurie et les médicaments abaissant le taux de lipides). Aussi, notre étude n’est pas enregistrée et ne comportait pas de protocole pré-étude.

**Conclusion:**

La supplémentation en vitamine D native est sûre et elle améliore le statut en vitamine D des patients atteints de MRD. La vitamine D semble modifier la protéinurie et le métabolisme lipidique en contexte de MRD, mais d’autres essais bien conçus et intégrant des traitements bien établis sont nécessaires. Globalement, il existe peu de données probantes sur les effets pléiotropiques bénéfiques de la vitamine D chez les patients atteints de MRD.

## Introduction

Diabetic kidney disease (DKD) affects approximately 1 in every 3 patients with diabetes mellitus and is the number one cause of chronic kidney disease (CKD) and end-stage kidney disease (ESKD). A high prevalence of vitamin D deficiency, characterized by low levels of 25-hydroxyvitamin D_3_ (25(OH)D_3_), has been repeatedly documented in patients with DKD, the rates of which exceed both those of healthy individuals and patients with diabetes but no kidney disease.^1-5^ Given that the incidence of T2D is rising globally,^6^ the use of vitamin D as a potential treatment to improve clinical outcomes in patients with DKD may have important public health implications.

In recent years, greater attention has been given to the role of native forms of vitamin D (eg, cholecalciferol, ergocalciferol, and calcifediol) in the management of patients with kidney disease.^7^ Conversion of 25(OH)D_3_ to calcitriol, the active form of vitamin D, by 1-alpha-hydroxylase (CYP27B1) in the kidneys is primarily responsible for its effects on circulating levels of minerals. However, this enzyme is also expressed in a variety of other tissues, including the pancreas, breast, colon, and parathyroid gland, supporting the potential for extrarenal vitamin D hydroxylation and possible benefits of native vitamin D even in patients with low kidney function.^7^ Therefore, vitamin D may have actions beyond its acknowledged role in mineral homeostasis with potential pleiotropic effects benefiting cardiovascular disease, host immunity, cancer incidence, glucose homeostasis, inflammation, and kidney disease progression.

Cardiovascular and kidney disease remain leading causes of morbidity and mortality in patients with T2D; thus, treatment strategies to improve these outcomes remain a major priority.

This heightened risk may be due, in part, to a higher burden of inflammation and endothelial dysfunction. In its early stages, DKD is characterized by glomerular hyperfiltration and microalbuminuria which can then progress to proteinuria, a central hallmark of the disease.^8^ In patients with DKD, low levels of 25(OH)D_3_ have been associated with increased urinary albumin-to-creatinine ratio (UACR)^2,9^ and higher levels of UACR predict adverse cardiovascular outcomes.^10,11^ Evidence suggests that in addition to predicting the progression to kidney failure and death, lowering proteinuria can slow kidney deterioration.^12-14^ A positive impact of vitamin D on proteinuria and/or cardiovascular risk appears plausible considering that vitamin D appears to have renoprotective effects such as downregulation of oxidative stress, anti-inflammatory and immunomodulatory signaling, suppression of renin expression, and prevention of extracellular matrix accumulation.^15^ However, studies examining the effects of vitamin D therapy in patients with DKD yield mixed results—some report reductions in proteinuria and/or inflammation,^16-20^ whereas others document no significant changes following treatment.^21-23^

The major activating enzyme in the vitamin D pathway, CYP27B1, is now known to be expressed throughout the body; therefore, native vitamin D may have effects on a range of systems beyond mineral homeostasis. The purpose of this review was to examine the randomized controlled trial (RCT) data examining the response of clinical outcomes and surrogate clinical and laboratory measures beyond mineral metabolism to native vitamin D supplementation in adults with DKD.

## Methods

The review was guided by the following research question: What is the impact of native vitamin D supplementation on clinical outcomes and surrogate clinical and laboratory measures in RCTs of patients with DKD? The corresponding PICOS criteria (Population, Intervention, Comparison, Outcomes, and Settings) are listed in Table 1.

**Table 1.** PICOS (Population, Intervention, Comparison, Outcome and Settings) Criteria for the Inclusion of Studies Evaluating the Effects of Native Vitamin D Supplementation.

Parameter	Inclusion criteria
Population	Adults with diabetic kidney disease
Intervention	Controlled vitamin D (native form)
Comparison	Nonexposed control group
Outcome	Clinical outcomes or surrogate laboratory measures
Settings	Randomized controlled trials

### Eligibility Criteria

Studies were required to be RCTs of native vitamin D supplementation in adults with DKD in which a clinical outcome and/or surrogate clinical or laboratory measure was examined. The initial literature search targeted the general population of patients with CKD. Based on the high volume of studies identified, eligible studies were subsequently categorized based on the type of kidney disease, with the present article tailored to DKD. Only intervention studies consisting of supplementation with a native vitamin D form (ergocalciferol, cholecalciferol, calcifediol) and a control or placebo comparison group were included. We considered studies reporting any clinical outcome or surrogate clinical or laboratory measure. Given our focus on pleiotropic effects of vitamin D, we excluded studies that only reported vitamin D status and mineral metabolism parameters. There were no requirements for vitamin D dosage or study duration.

### Information Sources and Search Strategy

A comprehensive search approach was employed to locate published studies and unpublished studies (gray literature) in the form of preprints, conference materials, and data from clinical trial registries. A preliminary search was conducted by a librarian in PubMed and Google Scholar, followed by an analysis of relevant studies, to identify applicable text words and database-specific subject headings. A comprehensive search strategy was developed in Embase (Ovid) and peer-reviewed by a second Librarian at the Health Sciences Library, Queen’s University. The final Embase strategy was adapted for MEDLINE (Ovid), EBM Reviews for Cochrane Central Register of Controlled Trials (CENTRAL via Ovid), Web of Science Core Collection, ProQuest Dissertations and Theses Global, and medRxiv. All adapted database search strategies underwent an independent peer review.

For the Embase search, the validated Cochrane Highly Sensitive Search Strategy for identifying controlled trials^24^ was used to search for RCTs given the high volume of results when combining search terms for the concepts of vitamin D and kidney disease. For the MEDLINE search, the Cochrane Highly Sensitive Search Strategy for identifying randomized trials in Ovid MEDLINE: sensitivity-maximizing version (2008 revision)^25^ was used, and a combination of the 2 database filters was adapted for searching in Web of Science. All databases were searched from inception to May 2, 2022, and no language or date restrictions were applied. An update was performed across all databases to retrieve records from this period up to January 31, 2023, which resulted in an additional 317 records for screening after duplicates were removed.

The number of search results from all databases and information sources searched after the January 31, 2023 update was 9632. The number of records after removing duplicates in Covidence systematic review software was 6518. The total number of records identified for each database and information source is provided in Appendix 1 (Supplementary File). Search strategies for each database and information source searched are presented in Appendix 2 (Supplementary File).

### Study Selection Process

Study selection was performed using the Covidence systematic review software. After studies were imported for screening, duplicates were removed automatically by the review system. Subsequently, all screening was performed manually by reviewers. Abstracts were each reviewed independently by 2 reviewers with disagreements resolved through discussion or with a third reviewer. All included abstracts progressed to full-text screening. Each full text was screened independently by 2 reviewers. Reviewers indicated why each full text was excluded using a prespecified list of options. Conflicts were again resolved through discussion or by a third reviewer. When abstracts had no corresponding full text, they were included only if there was sufficient detail in the reporting of the results. One abstract^21^ that had been initially excluded in the screening process was later rescreened and included on discovery of the full text.

### Data Collection and Synthesis

Study characteristics for each trial were synthesized: first author name, country, date of publication, participant age, intervention characteristics, inclusion criteria, primary outcomes examined, and the number of participants randomized versus included in final analysis. Funding sources were also noted. Results data were subsequently extracted, converted into SI units, and organized into tables manually. All data collection and synthesis was performed manually by one reviewer, for a total of 2 times, to ensure consistent results were obtained. Where results were unclear, a second reviewer examined the original publication. All clinical outcomes and surrogate clinical and laboratory measures reported in the trials are discussed in the results. The examined outcomes were proteinuria, measures of glucose and lipid metabolism, inflammation and oxidative stress, clinical cardiovascular parameters (eg, blood pressure, flow-mediated dilation [FMD]), kidney function biomarkers, and bone and fat mass. Although not considered outcomes, levels of 25(OH)D_3_ and calcium were reported for each trial.

### Effect Measures

The treatment effect was quantified by measurements taken at baseline and study end (or the change from baseline to study end) with corresponding tests of statistical significance between and/or within groups. One study^26^ only reported *P* values for the difference between groups at baseline and study end (rather than the between-group difference in the change from baseline) for certain outcomes so these values are denoted by an asterisk. Mean and standard deviation or median and interquartile range are presented. In the case where there were multiple manuscripts from the same trial, results are presented as a single category according to the first author’s last name(s) in the tables. Given the heterogeneity in study design and measurement of outcomes, no meta-analyses were conducted.

### Assessment of Risk of Bias

The revised Cochrane Risk of Bias Tool 2^27^ was used to evaluate bias. Two individuals independently reviewed and assigned scores to each study using manual templates. Conflicts were resolved by a third and, if needed, fourth reviewer until consensus was reached. Risk of bias assessments were performed for each report for trials with multiple publications but are combined into a single category in the bias assessment table.

## Results

### Study Selection

**Figure 1.**
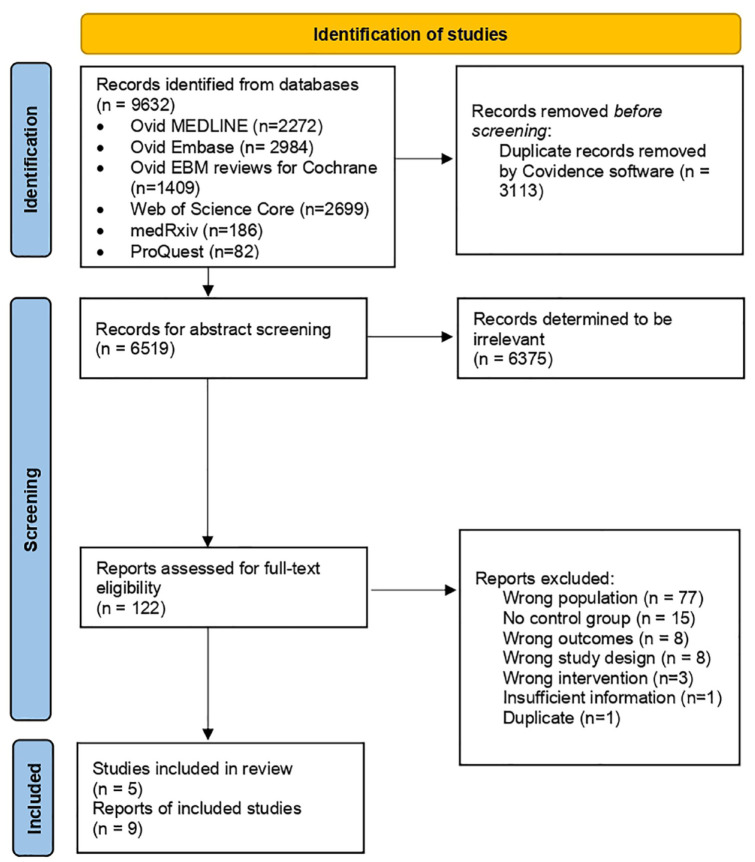
PRISMA (Preferred Reporting Items for Systematic Reviews and Meta-Analyses) systematic review flow diagram.

Figure 1 outlines the literature search process. In total, 3113 duplicates were removed from the initial 9632 studies identified by the search software. Of the remaining 6519 studies, 6375 were classified as irrelevant in abstract screening. In all, 122 studies subsequently underwent full-text review. A total of 9 publications were identified as eligible within the patient population of T2D and DKD, which corresponded to 5 different study cohorts.

### Study Characteristics

Table 2 displays the study characteristics of the included trials. All were single-center double-blinded RCTs conducted in Asia (Iran, Sri Lanka, India). A total of 377 participants (189 intervention and 188 controls) were enrolled, with a mean age ranging from approximately 40 to 60 years old. All trials were conducted in patients with T2D and a baseline UACR above a certain threshold level, most commonly 30 mg/g. Three trials required that participants have vitamin D insufficiency or deficiency at baseline. Every trial evaluated vitamin D_3_ (cholecalciferol) supplementation—4 in the form of oral capsules and 1 via intramuscular injection. Comparison groups all received placebo treatment. Vitamin D doses ranged from the equivalent of 20 000 to 200 000 IU per month. Study duration was 6 months or shorter, though 1 study with a 6-month duration^28^ conducted an additional 6-month follow-up in a randomly selected subgroup of the sample. Various laboratory and clinical endpoints were reported, most often as comparisons between values at baseline and study end for each group but occasionally as the difference between the intervention and control groups at each point. All studies appeared to use intention-to-treat analyses or modified intention-to-treat analyses, wherein only patients with outcomes were included.

**Table 2.** Study Characteristics of Included Trials.

First author, year, country	Inclusion criteria	Route	Intervention	Follow-up, mo	Randomized, n		Included in final analysis, n		Primary outcome(s)	Age (mean ± SD)	
					Vit D	Control	Vit D	Control		Vit D	Control
Ahmadi et al,^21^ 2013, Iran	UACR > 30 mg/g, 3 mos of ACEIs or ARBs, 25(OH)D <72.5 nmol/L	Oral	Vit D_3_; 50 000 IU/wk	3	30	30	28	23	UACR	58.32 ± 11.1	57.12 ± 10.17
Barzegari et al^29^ and Esfandiari et al,^30^ 2019, Iran	GFR <60 mL/min, albuminuria >30 mg/d, 25(OH)D 37-75 nmol/L	Oral	Vit D_3_; 5000 IU/wk	2	25	25	25	25	Metabolic profile, inflammation and oxidative stress	39.7 ± 7.3	43.7 ± 6.1
Liyanage et al^31,32^ 2017 and 2018, Sri Lanka	GFR >30 mL/min, UACR > 30 mg/g	IM	Vit D_3_; 50 000 IU/mo	6	43	42	41	41	CV risk profile, urine albumin	56 ± 10	59 ± 8
Liyanage et al^28^ 2021, Sri Lanka	GFR >30 mL/min, UACR > 30 mg/g	IM	Vit D_3_; 50 000 IU/mo	6^a^	43	42	39	38	BMD, BMC	56 ± 10	59 ± 8
Mahapatra et al^33^ 2020^b^ and Mahapatra et al^26^ 2021, India	eGFR ≥ 120 mL/min/1.73 m^2^ and any UACR or eGFR 60-120 mL/min/1.73 m^2^ and UACR 30-300 mg/g, no ACEIs or ARBs	Oral	Vit D_3_; 60 000 IU/wk × 4 wk, 60 000 IU/mo × 4 mo	6	61	61	54	49	Flow-mediated dilation, UAGT, UACR	48.74 ± 10.60	49.31 ± 10.49
Momeni et al,^34^ 2017, Iran	Proteinuria >150 mg/d, GFR > 50 mL/min^1^ or serum creatinine <177 μmol/L, 25(OH)D < 75 nmol/L	Oral	Vit D_3_; 50 000 IU/wk	2	30	30	29	28	Proteinuria (24 hours)	62.9 ± 9.3	62.4 ± 9

*Note.* UACR = urinary albumin-to-creatinine ratio; ACEIs = angiotensin-converting enzyme inhibitors; ARBs = angiotensin II receptor blockers; GFR = glomerular filtration rate; IM = intramuscular; CV = cardiovascular; BMD = bone mineral density; BMC = bone mineral content; UAGT = urinary angiotensinogen.

a An additional follow-up was conducted 6 months after treatment cessation for a total of 12 months since baseline.

b Abstract.

1 Equation for GFR not provided.

### Risk of Bias Assessment

Table 3 presents assessments of bias for the primary outcomes of each trial. There were concerns about selection of the reported results in all trials as there was no indication that a prespecified analysis plan was followed. The trial by Barzegari et al^29^ and Esfandiari et al^30^ was rated as high risk in this category due to evidence of selective reporting from multiple analyses of the data. Two trials^21,26,35^ received high-risk scores in the “effect of adhering to the intervention” and “missing outcome data” categories as there was little information on adherence and a significant proportion of withdrawals, which differed between groups. Two trials^21,34^ presented concerns in the randomization process and the effect of assignment to intervention as insufficient information was provided regarding allocation concealment and blinding.

**Table 3.** Risk of Bias Within Studies.

	Randomization process	Effect of assignment to intervention	Effect of adhering to intervention	Missing outcome data	Measurement of outcomes	Selection of the reported results
Ahmadi et al^21^	Some concerns	Some concerns	High	High	Low	Some concerns
Barzegari et al^29^ and Esfandiari et al^30^	Low	Low	Low	Low	Low	High
Liyanage et al^28,31,32^	Low	Low	Low	Low	Low	Some concerns
Mahapatra et al^26,33^	Low	Low	High	High	Low	Some concerns
Momeni et al^34^	Some concerns	Some concerns	Low	Low	Low	Some concerns

### Vitamin D Supplementation and Proteinuria

A measurement of proteinuria at baseline and study end was reported in each trial (Table 4). Barzegari et al^29^ and Esfandiari et al^30^ included patients with baseline albuminuria reported as >30 mg/d. They reported a reduction in proteinuria in the intervention group after 2 months. However, the change in proteinuria was reported in milligrams per deciliter, which does not account for urine volume, and the use of renin-angiotensin-aldosterone system (RAAS)-inhibiting medications by the participants was not reported. Liyanage et al^31^ reported similar results in a sample with initial UACR >30 mg/g where all trial participants were taking either an angiotensin-converting enzyme inhibitor (ACEI) or an angiotensin II receptor blocker (ARB). The UACR was reduced in the intervention group (*P* < .001). Mahapatra et al^26^ reported a reduction in UACR in the intervention group (*P* = .001) and an increase in the placebo group (*P* = .01). All participants had either (1) eGFR ≥120 mL/min/1.73 m^2^ and any UACR or (2) eGFR 60 to 120 mL/min/1.73 m^2^ and UACR 30 to 300 mg/g. This trial excluded patients taking ACEIs and/or ARBs. Momeni et al^34^ reported no change in proteinuria after 2 months in the intervention group but an increase in the controls; however, RAAS-inhibiting medication use was not reported. Conversely, Ahmadi et al^21^ reported no change in UACR in either group after 3 months. All participants had an initial UACR >30 mg/g. This was the only study where the between-group difference in the change in proteinuria from baseline was not significant. Notably, all participants enrolled in this study were required to have taken ACEIs or ARBs for 3 months prior.

**Table 4.** Vitamin D Supplementation and Proteinuria.

Author	Outcome metric	Study arms	N in final analysis	% on ACEI or ARBs	Outcome measurements		*P* value (within group)	*P* value (between group)
					BL	End		
Ahmadi et al^21^	UACR, mg/mmol	Vit D	28	100	13.63 ± 16.43	12.60 ± 14.58	.583	.844
		Placebo	23		10.79 ± 6.49	9.99 ± 7.45	.450	
Barzegari et al^29^, Esfandiari et al^30^	Proteinuria, g/L	Vit D	25	NR	3.33 ± 1.13	2.34 ± 1.19	**.001**	**.03**
		Placebo	25	NR	3.25 ± 1.11	3.20 ± 1.02	.72	
Liyanage et al^28,31,32^	UACR, mg/mmol	Vit D	41	100	19.14 ± 4.05	13.80 ± 6.15 *@3mo* 13.29 ± 5.11 *@6mo*	**<.001**	**.001**
		Placebo	41		21.00 ± 5.72	18.18 ± 7.16 *@3mo* 18.46 ± 6.35 *@6mo*	.06	
Liyanage et al^28,31,32^	UACR, mg/mmol	Vit D	54	0	5.88 (4.21-17.47)	4.41 (2.71-11.84)	**<.001**	**<.001**
		Placebo	49		7.19 (4.27-12.81)	6.78 (4.40-12.8)	**.01**	
Momeni et al^34^	Proteinuria, mg/d	Vit D	29	NR	962.62 ± 885.99	892.24 ± 879.40	.27	**.028**
		Placebo	28	NR	775.71 ± 640.94	971.60 ± 940.24	**.025**	

*Note.* No studies reported usage of other RAAS blockers. Data presented as mean ± SD or median (IQR). ACEI = angiotensin-converting enzyme inhibitor; ARB = angiotensin II receptor blocker; UACR = urinary albumin-to-creatinine ratio; RAAS = renin-angiotensin-aldosterone system; IQR = interquartile range NR = Not Reported; BL = Baseline. Bold values indicate *P* values with a significance level < 0.05.

### Vitamin D Supplementation and Measures of Lipid and Glucose Metabolism

Table 5 displays the results of 5 trials that examined measures of lipid and glucose metabolism in response to vitamin D treatment. Barzegari et al^29^ and Esfandiari et al^30^ reported a decrease in total cholesterol and low-density lipoprotein (LDL) and an increase in high-density lipoprotein (HDL) in the intervention group after 2 months but no changes in the placebo group. Triglycerides did not change in the intervention group but increased in the placebo group. This was the only trial to control for the effects of lipid-lowering medications as this was an exclusion criterion for participation. Liyanage et al^32^ reported reductions in total cholesterol and LDL and an elevation in HDL from baseline in the vitamin D group but no change in the placebo group. The decrease in triglycerides from baseline in the vitamin D group approached significance (*P* = .06), whereas levels remained stable in the placebo group (*P* = .62). Mahapatra et al^26^ reported no significant between-group difference in triglyceride levels at baseline or study end.

**Table 5.** Vitamin D Supplementation and Measures of Lipid and Glucose Metabolism.

Author	Outcome metric	Study arms	N in final analysis	% on drugs affecting lipids or glucose	Outcome measurements		*P* value (within group)	*P* value (between group)
					BL	End		
Ahmadi et al^21^	HbA_1c_, %	Vit D	28	66.7 (oral hypoglycemic drugs)	7.13 ± 1.33	7.22 ± 1.20	.636	.467
		Placebo	23		7.17 ± 1.4	7.09 ± 1.4	.688	
Barzegari et al^29^ and Esfandiari et al^30^	Total cholesterol, mg/dL	Vit D	25	0 (lipid-lowering drugs)	4.23 ± 1.2	3.60 ± 0.75	**.02**	NR
		Placebo	25		4.23 ± 0.88	4.60 ± 0.88	.12	NR
	LDL, mmol/L	Vit D	25		2.39 ± 0.98	1.83 ± 0.93	**.03**	NR
		Placebo	25		2.41 ± 0.65	2.63 ± 0.96	.31	NR
	HDL, mmol/L	Vit D	25		0.96 ± 0.19	1.06 ± 0.32	**.001**	NR
		Placebo	25		0.91 ± 0.19	0.92 ± 0.20	**.001**	NR
	TG, mmol/L	Vit D	25		1.94 ± 1.0	1.72 ± 0.69	.29	NR
		Placebo	25		1.98 ± 0.86	2.28 ± 0.86	**.02**	NR
	HbA_1c_, mmol/L	Vit D	25		6.36 ± 1.1	6.26 ± 1.6	.79	.66
		Placebo	25		6.61 ± 1.5	6.16 ± 1.4	.13	
	FBS, mmol/L	Vit D	25		7.17 ± 0.65	6.21 ± 0.75	**<.0001**	**<.0001**
		Placebo	25		7.21 ± 0.58	7.27 ± 0.65	.69	
	HOMA-IR	Vit D	25		11.50 ± 3.86	9.31 ± 2.97	**<.001**	**.001**
		Placebo	25		11.45 ± 3.41	11.64 ± 4.20	.74	
	Insulin, mIU/L	Vit D	25		36.11 ± 11.48	34.23 ± 11.26	.069	.27
		Placebo	25		35.78 ± 10.33	35.68 ± 10.80	.94	
Liyanage et al^28,31,32^	Total cholesterol, mmol/L	Vit D	41	NR	5.04 ± 0.78	4.95 ± 0.72 *@3mo* 4.80 ± 0.70 *@6mo*	**<.001**	.50
		Placebo	41	NR	5.03 ± 0.83	5.00 ± 0.80 *@3mo* 5.09 ± 0.81*@ 6mo*	.24	
	LDL, mmol/L	Vit D	41	NR	3.10 ± 0.74	2.99 ± 0.71 *@3mo* 2.74 ± 0.69 *@6mo*	**<.001**	.70
		Placebo	41	NR	3.02 ± 0.73	2.96 ± 0.74 *@3mo* 3.02 ± 0.78 *@3mo*	.34	
	HDL, mmol/L	Vit D	41	NR	1.30 ± 0.19	1.33 ± 0.18 *@3mo* 1.44 ± 0.18 *@6mo*	**<.001**	**<.001**
		Placebo	41	NR	1.38 ± 0.28	1.39 ± 0.28 *@3mo* 1.39 ± 0.25 *@6mo*	.40	
	TG, mmol/L	Vit D	41	NR	1.39 ± 0.47	1.38 ± 0.45 *@3mo* 1.33 ± 0.36 *@6mo*	.062	.44
		Placebo	41	NR	1.45 ± 0.57	1.44 ± 0.56 *@3mo* 1.45 ± 0.51 *@6mo*	.62	
	FBS, mmol/L	Vit D	41	NR	7.12 (0.75)	6.98 ± 0.74 *@3mo* 6.99 ± 0.60 *@6mo*	.08	.23
		Placebo	41	NR	7.23 (0.69)	7.25 ± 0.56 *@3mo* 7.09 ± 0.59 *@6mo*	**.02**	
	Plasma renin, pmol/L	Vit D	41	NR	0.35 ± 0.13	0.21 ± 0.11	**<.001**	**.006**
		Placebo	41	NR	0.36 ± 0.11	0.34 ± 0.11	**.02**	
Mahapatra et al^26,33^	TG, mmol/L	Vit D	54	NR	1.89 (1.31-2.41)	1.66 (1.31-2.28)	NR	.53*
		Placebo	49	NR	1.69 (1.29-2.02)	1.83 (1.38-2.20)	NR	
	HbA_1c_, %	Vit D	54	NR	7.37 ± 0.95	7.27 ± 1.27	NR	.32*
		Placebo	49	NR	7.19 ± 0.68	7.31 ± 0.79	NR	
	UAGT, ng/mL	Vit D	54	NR	27.69 (16.35-36.85)	13.75 (7.97-18.75)	**<.001**	**<.001**
		Placebo	49	NR	13.70 (8.30-25.23)	18.70 (14.35-27.45)	**<.01**	
Momeni et al^34^	HbA_1c_, %	Vit D	29	NR	7.82 ± 0.86	8.02 ± 1.23	.259	.406
		Placebo	28	NR	8.14 ± 1.26	8.10 ± 0.96	.895	
	FBS, mmol/L	Vit D	29	NR	8.13 ± 2.94	8.48 ± 2.74	.482	.544
		Placebo	28	NR	8.41 ± 2.91	8.60 ± 4.02	.882	

*Note.* Data presented as mean ± SD or median (IQR). HbA_1c_ = glycosylated hemoglobin; LDL = low-density lipoprotein; HDL = high-density lipoprotein; TG = triglycerides; FBS = fasting blood sugar; UAGT = urinary angiotensinogen; IQR = interquartile range; HOMA-IR = Homeostatic Model Assessment for Insulin Resistance; NR = Not reported; BL = Baseline. Bolded values are to imply significant results.

* *P* value comparing cholecalciferol and placebo group at 6-month time point.

Barzegari et al^29^ and Esfandiari et al^30^ reported a decreased fasting blood sugar (FBS) and Homeostatic Model Assessment for Insulin Resistance (HOMA-IR) in the vitamin D group at study end, as well as a reduction in insulin levels that approached significance (*P* = .07). The controls exhibited no significant changes. Conversely, Liyanage et al^28^ reported a reduction of FBS in the placebo group at 6 months, but the decrease in the intervention group did not reach significance (*P* = .08). Momeni et al^34^ documented no changes in FBS in either group. The 4 studies that measured glycosylated hemoglobin reported no change in either group. The trial by Ahmadi et al, for which glycosylated hemoglobin was the only measured parameter of glucose metabolism, was the only one to report the use of glucose-lowering medications: 66.7% of their sample was receiving oral hypoglycemic drugs.

Liyanage et al^31^ also found a significant reduction in plasma renin from baseline in both the treatment (*P* < .001) and control (*P* = .02) groups after 6 months. In the study that measured urinary angiotensinogen,^26^ there was a reduction in the vitamin D group from baseline and an elevation in the placebo group.

### Vitamin D Supplementation and Inflammation and Oxidative Stress

Barzegari et al^29^ and Esfandiari et al^30^ examined markers of inflammation and oxidative stress (Supplementary Table S1). There were no changes in total antioxidant capacity, superoxide dismutase, glutathione peroxidase, and catalase levels from baseline to the 2-month follow-up in either group. Malondialdehyde levels increased significantly in the placebo group but not in the intervention group compared with baseline. Tumor necrosis factor α and interleukin-6 significantly decreased in the intervention group.

### Vitamin D Supplementation and Clinical Cardiovascular Parameters

Liyanage et al^32^ reported unchanged and decreased systolic blood pressure (SBP) and diastolic blood pressure (DBP), respectively, at 6 months compared with baseline in the intervention group (Supplementary Table S2). The placebo group demonstrated elevations in SBP and DBP. Ahmadi et al^35^ reported decreased SBP in both groups after the 3-month study compared with baseline. Mahapatra et al^33^ reported significantly higher median FMD at 6 months in the intervention group compared with the placebo group, despite similar between-group values at baseline.

### Vitamin D Supplementation and Kidney Function

There were mixed findings regarding kidney function (Supplementary Table S3). Three studies^21,26,29^ reported no change in estimated glomerular filtration rate (eGFR) or serum creatinine from baseline to study end in either group. In the trial using intramuscular injection,^31^ there was a decrease in serum creatinine and increase in eGFR in the intervention group at 6 months compared with baseline but no changes in the placebo group. Ahmadi et al^21^ also documented reductions in blood urea nitrogen from baseline in the intervention group but no changes in the placebo group at 3 months.

### Vitamin D Supplementation and Bone and Fat Mass

One study^28^ examined bone and fat mass (Supplementary Table S4). Bone mineral density (BMD), bone mineral content (BMC), spine BMD, femoral neck BMD, and hip BMD were increased in the vitamin D group at 6 months compared with baseline. The elevation in trochanter BMD approached significance (*P* = .07). In the placebo group, there were no changes in these parameters but the decrease in BMC from baseline was almost significant (*P* = .07). No changes in total fat mass and lean mass were observed in either group. An additional follow-up at 6 months following treatment cessation (12 months from baseline) in 25 participants selected randomly from each group reported significant reductions in total BMD and BMC, but not regional BMD, compared with the 6-month time point in the vitamin D group. Values in the control group did not change between 6 and 12 months. Total fat mass and lean mass did not change in either group.

### 25(OH)D and Calcium Levels

All 5 trials reported an increase in serum 25(OH)D levels from baseline to study end in the intervention group (Supplementary Table S5). In 3 of these, the control group experienced either a reduction or a nonsignificant change in 25(OH)D from baseline.^21,26,31^ In the trial reported by Momeni et al, the control participants also had higher 25(OH)D levels at study end but the increase in the vitamin D–treated group was greater.^34^ In the trial by Barzegari et al^29^ and Esfandari et al,^30^ 25(OH)D levels increased in both groups.

There were mixed findings regarding calcium. In the 3-month study^21^ using 50 000 IU/wk of oral vitamin D_3_, serum calcium increased in both groups from baseline to study end. In the 2-month study using 5000 IU/wk vitamin D_3_,^29,30^ there was no change in serum calcium from baseline in either group. In a 6-month study,^26^ serum calcium was similar between groups at baseline but higher in the vitamin D group at study end.

### Vitamin D Supplementation and Reporting of Adverse Effects

Three of the 5 trials^28-32,35^ reported no adverse effects throughout the study period. In 1 trial,^26^ 2 participants in the placebo group and 3 in the intervention group experienced self-limiting nausea which did not require discontinuation. There were no major adverse events in either group. One study^34^ did not provide information about adverse effects.

## Discussion

Our systematic review is the first to examine trials evaluating vitamin D supplementation in patients with DKD that focuses on the potential pleiotropic benefits beyond mineral homeostasis. To date, no trial has been conducted in patients with DKD that addresses clinical events or mortality. Overall, the trials were small and heterogeneous regarding study design with limited power to address the outcomes. Despite these limitations, there were trends to suggest that vitamin D_3_ may lead to a reduction in proteinuria and total and LDL cholesterol. However, these observed trends are weakened substantially by the limited reporting on the use of drugs known to alter proteinuria and cholesterol. Whether native vitamin D has an effect beyond established treatments is unknown but a potential topic of future interest. Based on sparse and sometimes conflicting data, there is insufficient evidence to suggest a beneficial effect of native vitamin D on glucose metabolism, inflammation, kidney function or bone disease in patients with DKD.

Vitamin D_3_ is the recommended treatment for vitamin D deficiency according to the National Osteoporosis Foundation,^36^ National Osteoporosis Society,^37^ and the International Osteoporosis Foundation (IOF),^38^ among others. Vitamin D status improved in the intervention group compared with the placebo group in 4 of the 5 trials examined in our review and achieved levels generally exceeded 75 nmol/L, the Endocrine Society definition for vitamin D sufficiency. It is unlikely, therefore, that null findings were based on not achieving adequate 25(OH)D levels.

The most frequently studied outcome in the trials was proteinuria. Four of 5 trials reported significant between-group differences in the change in proteinuria from baseline to study end, supporting a potential benefit of vitamin D. However, only 3 trials reported the use of ACEIs and ARBs, first-line drug classes that decrease proteinuria and limit progression of DKD.^39^ Specifically, Mahapatra et al^26^ excluded participants taking ACEIs and ARBs, while in the trials by Ahmadi et al^21^ and Liyanage et al,^31^ all participants were receiving either ACEIs or ARBs at baseline. There was no clear signal between these enrollment criteria and proteinuria outcomes as both Mahapatra et al and Liyanage et al reported greater proteinuria reduction in the vitamin D than in the placebo group. The trial by Ahmadi et al was the only one to report no change in either group.

Proteinuria reduction, as well as cardiovascular risk reduction, is typically accomplished through the use of agents that block the RAAS and, more recently, with the use of sodium-glucose cotransporter 2 (SGLT2) inhibitors.^8^ In a post hoc analysis of the CREDENCE trial where all patients had T2D and were taking RAAS blockade, albuminuria was decreased by 31% at week 26 in participants randomized to canagliflozin compared with placebo.^40^ The importance of this reduction in proteinuria was highlighted by the finding that each 30% reduction in UACR, relative to placebo, was associated with a 29% lower risk of the composite outcome of doubling of serum creatinine, ESKD, or death due to renal causes. The time frame over which the included studies were conducted likely predated the routine use of SGLT2 inhibitors. However, none of the examined trials reported the use of aldosterone antagonists such as spironolactone, another class of RAAS blockers. Mineralocorticoid receptor antagonism also confers an additional reduction in albuminuria in patients with CKD already treated with an RAS-inhibitor but may increase the risk of adverse events.^41^ Thus, future research would be required to elucidate any interactions between vitamin D and both RAAS and SGLT2 blockade in patients with DKD. Finally, it should also be noted that treatments that are kidney protective also lower proteinuria; however, the Food and Drug Administration does not yet accept albuminuria as a valid surrogate for delaying progression of CKD.

Albuminuria is a modifiable therapeutic target in the population with T2D but whether vitamin D provides any benefit either alone or synergistically with more established treatments is not known. Research in streptozotocin-treated diabetic mice has demonstrated that combination therapy with paricalcitol (an analogue of the active form of vitamin D_2_) and the ARB losartan leads to greater preservation of kidney function and complete prevention of albuminuria.^42^ Furthermore, administration of paricalcitol has been found in 2 randomized placebo-controlled double-blinded trials to reduce albuminuria in patients already taking ACEIs or ARBs.^43,44^ One of these trials only enrolled patients with T2D, while the prevalence of T2D in the other trial was 70%. It has been hypothesized that these synergistic effects result from vitamin D–mediated suppression of the renin induction typically accompanying RAAS inhibitor use.^45^ The one trial that examined plasma renin levels did demonstrate a significant between-group difference in renin suppression with vitamin D treatment. All participants in this trial were taking RAAS blockers and the decrease in ACR observed correlated with the percentage change in plasma renin levels. Such findings are very preliminary but potentially important as even small additional reductions in proteinuria resulting from native vitamin D could have clinical relevance. For instance, a 2015 meta-analysis of 21 clinical trials reported a 23.7% reduction in ESKD risk for every 30% decrease in albuminuria, which was independent of the drug category as well as patient and trial characteristics.^46^

Two trials examined cholesterol levels, both reporting decreases in total cholesterol and LDL in the intervention group but not in the controls by study end.^29,32^ One of these trials also reported an increase in HDL in the intervention group and no change in the control group,^32^ while the other documented increases in HDL for both groups.^20^ However, it is a major limitation of these studies that cholesterol-lowering medication was not consistently reported. A 2016 meta-analysis of 17 RCTs that included only patients with T2D similarly reported significant reductions in serum total cholesterol and LDL following vitamin D treatment, but negligible changes in HDL and triglycerides.^47^ It is not entirely clear how vitamin D affects lipid levels, but effects on intestinal cholesterol absorption or de novo cholesterol production have been proposed.^47^ Hypotheses have also been generated from basic research. For instance, vitamin D has been found to reduce uptake of acetylated or oxidized LDL and subsequent foam cell formation in macrophages of patients with type 2 diabetes, with deletion of the vitamin D receptor increasing the speed of foam cell formation.^48^ Moreover, incubation of adipocytes with calcitriol has been shown to upregulate lipoprotein lipase gene expression, which could allow for greater clearance of lipoproteins from the circulation.^49^

Of the 4 trials that measured parameters of glucose metabolism, improvements were only documented in 1 trial, which reported reductions in FBS and HOMA-IR in the intervention but not in the placebo group. These data are difficult to interpret in the absence of adequate reporting of glucose-lowering treatment. Although vitamin D has been proposed as important for pancreatic beta-cell function and insulin sensitivity,^50^ there is limited supporting evidence in the literature. A 2017 systematic review of 29 trials investigating vitamin D supplementation in adults with T2D reported no impact on fasting blood glucose and only a moderate reduction in glycosylated hemoglobin.^51^ Furthermore, a systematic review and meta-analysis that examined vitamin D_3_ supplementation found no significant impact on insulin resistance, insulin secretion, glycosylated hemoglobin, or the prevention of T2D.^52^

Of the 2 studies that measured blood pressure, the reduction in SBP and DBP was only more significant than that of the placebo group in 1 study.^32^ Despite evidence for a role of vitamin D in RAAS axis regulation, there is presently limited evidence from randomized clinical trials and meta-analyses in human subjects to support a blood pressure–lowering effect of vitamin D.^53-55^

One study examined in our review reported an increase in FMD in the vitamin D group. This finding is consistent with reports that vitamin D deficiency is linked to reduced FMD in patients with CKD without diabetes^56^ and that supplementation with vitamin D_3_ improves FMD following a 16-week course of treatment.^57,58^ The mechanisms through which vitamin D could improve vascular endothelial function are unclear but may involve effects on inflammation or the lipid profile.^56^

One trial^29,30^ examined the effect of native vitamin D on inflammation and oxidative stress. The key findings were an increase in malondialdehyde in the placebo group and decreases in tumor necrosis factor α and interleukin-6 in the intervention group. Negative associations between vitamin D and inflammatory markers have been documented in the literature, but the directionality of this relationship is unclear.^59^ A 2015 systematic review examined 39 RCTs using vitamin D supplementation, reporting significant reductions in inflammatory markers in 17 of the trials. They observed almost no effects in samples of healthy subjects or in patients with stable cardiovascular disease, T2D, or obesity. Benefits were greatest in the setting of low baseline vitamin D levels or conditions associated with substantial inflammation.^59^ Considering that inflammatory signaling has been proposed as a mechanism of kidney microvascular damage and that low-grade inflammatory responses have been identified in the pathogenesis of DKD, the effects of vitamin D on inflammation could have a meaningful therapeutic role.^60^ There is limited evidence regarding the effects of vitamin D on oxidative stress; however, research in cultures treated with hydrogen peroxide has shown a reduction of apoptosis pathways in the presence of vitamin D receptor ligand.^61^

Our review showed no clear benefit of vitamin D on kidney function, though improvements in serum creatinine and eGFR were documented in the trial using intramuscular injections.^31^ Ahmadi et al also reported reductions in blood urea nitrogen in the intervention group.^21^ To date, there has been limited investigation of the relationship between native vitamin D treatment and kidney outcomes. One study in adults with prediabetes showed no effect of vitamin D_3_ supplementation on the change in eGFR or KDIGO risk scores from baseline.^62^ Nevertheless, if a proteinuria-lowering role of native vitamin D exists, as previously discussed, well-conducted research examining the long-term effects of vitamin D on kidney outcomes may be warranted.

One of the trials reported improvements in BMD and BMC in the vitamin D group, which were diminished 6 months after treatment cessation.^28^ In general, vitamin D deficiency is associated with secondary hyperparathyroidism, increased bone turnover, and decreased BMD.^63^ Several trials have examined the effect of vitamin D supplementation on bone parameters, generally in conjunction with calcium administration.^63^ A 2014 systematic review and meta-analysis of 23 randomized trials involving vitamin D_3_ or D_2_ supplementation (12 of which involved co-administration of calcium) reported that calcium administration did not affect outcomes and observed only a small benefit for BMD at the femoral neck.^64^ Whether individuals with DKD, who have high rates of vitamin D deficiency, may derive greater benefit from supplementation has not been adequately studied.

Certain limitations of our review must be acknowledged. Only data from 5 different cohorts were available from RCTs, with no more than 61 individuals per treatment arm enrolled in each study. All trials were conducted in Asian countries; therefore, the findings may not apply to all racial groups. All trials were conducted in single centers limiting generalizability within their own regions. Furthermore, some laboratory or clinical outcomes such as BMD were only examined in 1 or 2 trials, limiting the ability to draw definitive conclusions. Moreover, the follow-up periods were relatively short so whether the observed changes can persist with continued supplementation and modify a disease course with a long trajectory is unknown. Finally, the lack of information on concomitant medication use in most studies makes it impossible to isolate the pleiotropic effects of vitamin D—particularly those related to the lipid profile and proteinuria. Our investigation is also limited by the absence of review registration and a prestudy protocol.

All included trials had randomized double-blinded placebo-controlled designs, suggesting a greater degree of internal validity. However, in some cases, interpretation was limited by incorrect data or units, typographical errors and/or insufficient information regarding inclusion criteria, and the measurement of outcomes which is reflected in the bias assessments. In addition, the use of varying methods to measure outcomes (particularly proteinuria where it was reported as either UACR, a concentration in grams per liter, or 24-hour excretion) between trials limited the ability to observe trends across studies or conduct meta-analyses. Clear and consistent reporting of results should be prioritized in future RCTs.

Overall, there is a paucity of well-conducted clinical trials examining the effect of native vitamin D on potential pleiotropic clinical outcomes or surrogate laboratory measures. Vitamin D appears to be safe and well-tolerated but whether it improves a range of outcomes beyond mineral homeostasis in patients with DKD is unknown. Tempered by the significant limitations of these trials, our results suggest a potential impact of vitamin D_3_ on proteinuria and lipid metabolism with a limited associated risk of adverse events. However, a far greater body of evidence from research in larger samples with strict methods to control for concomitant drug use would be necessary to inform this field.

## Supplemental Material

sj-docx-1-cjk-10.1177_20543581231212039 – Supplemental material for Are There Any Pleiotropic Benefits of Vitamin D in Patients With Diabetic Kidney Disease?: A Systematic Review of Randomized Controlled Trials Click here for additional data file. Supplemental material, sj-docx-1-cjk-10.1177_20543581231212039 for Are There Any Pleiotropic Benefits of Vitamin D in Patients With Diabetic Kidney Disease?: A Systematic Review of Randomized Controlled Trials by Jaya K. Sharma, Sono Khan, Tristin Wilson, Nathan Pilkey, Sanjana Kapuria, Angélique Roy, Michael A. Adams and Rachel M. Holden in Canadian Journal of Kidney Health and Disease

sj-docx-2-cjk-10.1177_20543581231212039 – Supplemental material for Are There Any Pleiotropic Benefits of Vitamin D in Patients With Diabetic Kidney Disease?: A Systematic Review of Randomized Controlled Trials Click here for additional data file. Supplemental material, sj-docx-2-cjk-10.1177_20543581231212039 for Are There Any Pleiotropic Benefits of Vitamin D in Patients With Diabetic Kidney Disease?: A Systematic Review of Randomized Controlled Trials by Jaya K. Sharma, Sono Khan, Tristin Wilson, Nathan Pilkey, Sanjana Kapuria, Angélique Roy, Michael A. Adams and Rachel M. Holden in Canadian Journal of Kidney Health and Disease
