# Supplementary material for: Are There Any Pleiotropic Benefits of Vitamin D in Patients With Diabetic Kidney Disease? A Systematic Review of Randomized Controlled Trials
Source: Can J Kidney Health Dis. 2023 Nov 28;10:20543581231212039. doi: 10.1177/20543581231212039 (PMC10683388; doi:10.1177/20543581231212039)
Supplement: sj-docx-2-cjk-10.1177_20543581231212039 – Supplemental material for Are There Any Pleiotropic Benefits of Vitamin D in Patients With Diabetic Kidney Disease?: A Systematic Review of Randomized Controlled Trials [file sj-docx-2-cjk-10.1177_20543581231212039.docx]

**Supplementary File.**

**Table S1.** Vitamin D supplementation and inflammation and oxidative stress.

| Author | Outcome metric | Study arms | N included in final analysis | Outcome measurements | | *p* value (within group) | *p* value (b/w group) |
| --- | --- | --- | --- | --- | --- | --- | --- |
|  |  |  |  | BL | End |  |  |
| Barzegari, Esfandiari | TAC (mmol/L) | Vit D | 25 | 1.4 ± 0.46 | 1.6 ± 0.75 | 0.15 | NR |
|  |  | Placebo | 25 | 1.5 ± 0.44 | 1.5 ± 0.43 | 0.86 | NR |
|  | SOD  (U/g Hb) | Vit D | 25 | 1318.5 ± 228 | 1356 ± 189 | 0.62 | NR |
|  |  | Placebo | 25 | 1322.4 ± 197 | 1327.5 ± 178 | 0.44 | NR |
|  | GPX  (U/g Hb) | Vit D | 25 | 36.8 ± 8.7 | 37.7 ± 9.4 | 0.77 | NR |
|  |  | Placebo | 25 | 31.63 ± 6.9 | 30.6 ± 6.1 | 0.22 | NR |
|  | CAT  (units/mg protein) | Vit D | 25 | 68.9 ± 15 | 73.5 ± 15 | 0.12 | NR |
|  |  | Placebo | 25 | 68.6 ± 15 | 68.0 ± 15 | 0.36 | NR |
|  | MDA (mmol/L) | Vit D | 25 | 1.4 ± 0.4 | 1.6 ± 0.6 | 0.18 | NR |
|  |  | Placebo | 25 | 1.5 ± 0.3 | 1.6 ± 0.4 | **0.02** | NR |
|  | TNF-alpha (pg/mL) | Vit D | 25 | 136.6±33.65 | 118.47±27.58 | **0.002** | 0.34 |
|  |  | Placebo | 25 | 133.22±29.32 | 129.57±22.51 | 0.43 |  |
|  | IL-6 (pg/mL) | Vit D | 25 | 114.38±33.17 | 103.28±25.07 | **0.037** | 0.122 |
|  |  | Placebo | 25 | 113.17±27.17 | 110.76±20.13 | 0.53 |  |

Abbreviations: TAC- total antioxidant capacity, SOD- superoxide dismutase, GPX- glutathione peroxidase, CAT- catalase, MDA- malondialdehyde, TNF- tumor necrosis factor, IL- interleukin. Data presented as mean± SD.

**Table S2.** Vitamin D supplementation and cardiovascular outcomes.

| Author | Outcome metric | Study arms | N included in final analysis | Outcome measurements | | *p* value (within group) | *p* value (b/w group) |
| --- | --- | --- | --- | --- | --- | --- | --- |
|  |  |  |  | BL | End |  |  |
| Ahmadi | SBP (mmHg) | Vit D | 28 | 125.35±15.51 | 119.67±16.74 | **0.033** | NS |
|  |  | Placebo | 23 | 122.86±12.8 | 118.17±17.25 | **0.022** |  |
| Liyanage | SBP (mmHg) | Vit D | 41 | 120±8 | 120±8 *@3mo*  121±7 *@6mo* | 0.59 | 0.07 |
|  |  | Placebo | 41 | 121±7 | 121±8 *@3mo*  127±6 *@6mo* | **<0.001** |  |
|  | DBP (mmHg) | Vit D | 41 | 71±6 | 69±6 *@3mo* 68±6*@6mo* | **<0.001** | 0.17 |
|  |  | Placebo | 41 | 70±6 | 72±6 *@3mo*  72±6 *@ 6mo* | **<0.001** |  |
| Mahapatra | Flow mediated dilation (%) | Vit D | 41 | NR | 24 (21.75-26.0) | N/A | **0.01*** |
|  |  | Placebo | 41 | NR | 22 (18-24) | N/A |  |

Abbreviations: SBP- systolic blood pressure, DBP- diastolic blood pressure, NS- not significant (specific p value not available). Data presented as mean± SD. *Comparison between vitamin D and placebo groups after six months.

**Table S3.** Vitamin D supplementation and kidney function.

| Author | Outcome metric | Study arms | N included in final analysis | Outcome measurements | | *p* value (within group) | *p* value (b/w group) |
| --- | --- | --- | --- | --- | --- | --- | --- |
|  |  |  |  | BL | End |  |  |
| Ahmadi | Blood urea nitrogen (mmol/L) | Vit D | 28 | 11.16 ± 3.8 | 10.42 ± 1.4 | **0.036** | 0.214 |
|  |  | Placebo | 23 | 11.24± 4.4 | 10.85± 3.2 | 0.589 |  |
|  | GFR (ml/min/1.73^2^) | Vit D | 28 | 71.15±19.77 | 72.00±19.63 | 0.734 | 0.482 |
|  |  | Placebo | 23 | 70.74±23.28 | 73.18±19.68 | 0.380 |  |
|  | Serum creatinine (umol/L) | Vit D | 28 | 98.1±26 | 96.4±19 | 0.702 | 0.251 |
|  |  | Placebo | 23 | 106±28 | 91.9±20 | 0.100 |  |
| Barzegari & Esfandiari | GFR (ml/min/1.73^2^) | Vit D | 25 | 45.87±17.39 | 46.96 ± 12.17 | 0.58 | 0.96 |
|  |  | Placebo | 25 | 46.97±12.26 | 46.46 ± 13.64 | 0.89 |  |
|  | Serum creatinine (umol/L) | Vit D | 25 | 116±29 | 100±58 | 0.95 | 0.25 |
|  |  | Placebo | 25 | 102±32 | 116±47 | 0.35 |  |
| Liyanage | Serum creatinine (umol/L) | Vit D | 41 | 76±11 | 71±11@3mo  68 ±10 @6mo | **<0.001** | 0.10 |
|  |  | Placebo | 41 | 77±20 | 77±18@3mo  77±18 @6mo | 0.84 |  |
|  | GFR (mL/min) | Vit D | 41 | 86.7±14.6 | 90.7±14.8@3mo  93.7±14.1@6mo | **<0.001** | **0.03** |
|  |  | Placebo | 41 | 83.2 ±16.1 | 83.4±15.6@3mo  83.9 ±14.9@6mo | 0.74 |  |
| Mahapatra | eGFR (mL/min) | Vit D | 54 | 104.5 (76.75-134.25) | 106 (77-121.5) | 0.24 | 0.41 |
|  |  | Placebo | 49 | 97 (74.5-122.0) | 100 (71.5-124.5) | 0.08 |  |
|  | Serum creatinine (umol/L) | Vit D | 54 | 61 (53-78) | 61 (53-78) | NR | 0.89* |
|  |  | Placebo | 49 | 70 (53-78) | 70 (53-78) | NR |  |

Abbreviations: GFR-glomerular filtration rate. Data presented as mean± SD or median (IQR). *p value comparing cholecalciferol and placebo group at 6-month time point.

**Table S4.** Vitamin D supplementation and bone and fat mass.

| Author | Outcome metric | Study arms | N included in final analysis | Outcome measurements | | *p* value (within group) | *p* value (b/w group) |
| --- | --- | --- | --- | --- | --- | --- | --- |
|  |  |  |  | BL | End |  |  |
| Liyanage (2021) | BMD (g/cm^2^) | Vit D | 39 | 1.038 ±0.120 | 1.059 ±0.107 | **0.01** | 0.61 |
|  |  | Placebo | 38 | 1.038 ±0.121 | 1.031 ±0.191 | 0.75 |  |
|  | BMC (g) | Vit D | 39 | 1757.95 ±383.68 | 1795.85 ±373.27 | **0.007** | 0.73 |
|  |  | Placebo | 38 | 1775.63 ±412.76 | 1721.64 ±369.70 | 0.074 |  |
|  | Spine BMD (g/cm^2^) | Vit D | 39 | 0.845 ±0.153 | 0.860 ±0.142 | **0.04** | 0.72 |
|  |  | Placebo | 38 | 0.848 ±0.132 | 0.836 ±0.119 | 0.27 |  |
|  | Femoral neck BMD (g/cm^2^) | Vit D | 39 | 0.731 ±0.153 | 0.746 ±0.142 | **0.03** | 0.43 |
|  |  | Placebo | 38 | 0.722 ±0.109 | 0.712 ±0.094 | 0.23 |  |
|  | Trochanter BMD (g/cm^2^) | Vit D | 39 | 0.615 ±0.111 | 0.627 ±0.103 | 0.07 | 0.46 |
|  |  | Placebo | 38 | 0.607 ±0.089 | 0.604 ±0.08 | 0.5 |  |
|  | Hip BMD (g/cm^2^) | Vit D | 39 | 0.876 ±0.148 | 0.899 ±0.149 | **0.008** | 0.25 |
|  |  | Placebo | 38 | 0.857 ±0.113 | 0.852 ±0.105 | 0.56 |  |
|  | Total fat mass (kg) | Vit D | 39 | 17.41 ±5367 | 18.21 ±5.56 | 0.06 | 0.20 |
|  |  | Placebo | 38 | 15.85 ±6.67 | 16.48 ±6.16 | 0.20 |  |
|  | Lean mass (kg) | Vit D | 39 | 38.92 ±8.32 | 39.64 ±7.73 | 0.09 | 0.16 |
|  |  | Placebo | 38 | 37.04 ±6.94 | 36.98 ±6.38 | 0.86 |  |

Abbreviations: BMD- bone mineral density, BMC- bone mineral content. Data presented as mean± SD.

**Table S5.** Levels of 25(OH)D and calcium.

| Author | Outcome metric | Study arms | N in final analysis | Outcome measurements | | *p* value (within group) | *p* value (b/w group) |
| --- | --- | --- | --- | --- | --- | --- | --- |
|  |  |  |  | BL | End |  |  |
| Ahmadi | 25(OH)D (nmol/L) | Vit D | 28 | 35.09±19.4 | 177.8±66.2 | **0.001** | **0.001** |
|  |  | Placebo | 23 | 40.12±15.2 | 44.07±46.3 | 0.10 |  |
|  | Calcium (mmol/L) | Vit D | 28 | 2.30±0.11 | 2.42±0.08 | **0.001** | 0.147 |
|  |  | Placebo | 23 | 2.35±0.08 | 2.44±0.10 | **0.002** |  |
| Barzegari, Esfandiari | 25(OH)D (nmol/L) | Vit D | 25 | 54.09±14.0 | 93.92±19.3 | **0.001** | NR |
|  |  | Placebo | 25 | 55.81±14.2 | 60.18±19.0 | **0.001** |  |
|  | Calcium (mmol/L) | Vit D | 25 | 2.07±0.26 | 2.14±0.29 | 0.40 | 0.14 |
|  |  | Placebo | 25 | 2.00±0.27 | 2.12±0.28 | 0.10 |  |
| Liyanage | 25(OH)D (nmol/L) | Vit D | 41 | 56.11±12.95 | 81.75±15.03 | **<0.001** | **<0.001** |
|  |  | Placebo | 41 | 49.64±16.46 | 45.67±17.20 | **0.004** |  |
| Mahapatra | 25(OH)D (nmol/L) | Vit D | 54 | 45.7  (35.9-61.1) | 61.02(49.42-75.17) | **<0.001** | **<0.001** |
|  |  | Placebo | 49 | 48.2  (38.9-60.9) | 46.42 (36.94-57.16) | **0.03** |  |
|  | Calcium (mmol/L) | Vit D | 54 | 2.28±0.15 | 2.40±0.16 | NR | **<0.01*** |
|  |  | Placebo | 49 | 2.25±0.13 | 2.15±0.17 | NR |  |
| Momeni | Vitamin D (nmol/L) | Vit D | 29 | 36.76±19.16 | 89.44 ± 34.35 | **0.0001** | **<0.001** |
|  |  | Placebo | 28 | 32.19±17.76 | 38.02 ± 23.90 | **0.02** |  |

Data presented as mean± SD or median (IQR) unless otherwise indicated. *p value comparing intervention and placebo group at 6-month time point. ^**^Differences were significant at baseline.

Appendix 1: Total number of records identified for each database and information source up to May 2, 2022 with update performed to include up to January 31, 2023.

| Ovid MEDLINE | 2204 |
| --- | --- |
| *May 2, 2022-Jan 31, 2023 Update* | 68 |
| Ovid Embase | 2786 |
| *May 2, 2022-Jan 31, 2023 Update* | 198 |
| Ovid EBM Reviews for Cochrane CENTRAL | 1321 |
| *May 2, 2022-Jan 31, 2023 Update* | 88 |
| Web of Science Core Collection | 2581 |
| *May 2, 2022-Jan 31, 2023 Update* | 118 |
| medRxiv | 151 |
| *May 2, 2022-Jan 31, 2023 Update* | 35 |
| ProQuest Dissertations and Theses Global | 82 |
| *May 2, 2022-Jan 31, 2023 Update* | 0 |
| Total number of records | 9632 |
| Total number of records after removing duplicates in Covidence | 6518 |

Appendix 2: Search Strategy Embase, MEDLINE, Cochrane CENTRAL, Web of Science, ProQuest Dissertations and Theses, medRxiv

**Embase Classic+Embase <1947 to 2022 May 02>**

1 chronic kidney failure/ or "chronic kidney disease-mineral and bone disorder"/ or renal osteodystrophy/ (131189)

2 end stage renal disease/ (43428)

3 hemodialysis/ or continuous hemodialysis/ or home dialysis/ (128603)

4 kidney graft/ (50037)

5 glomerulonephritis/ or acute glomerulonephritis/ or allergic glomerulonephritis/ or chronic glomerulonephritis/ or experimental glomerulonephritis/ or experimental autoimmune glomerulonephritis/ or focal glomerulonephritis/ or immunoglobulin a nephropathy/ or immunoglobulin m nephropathy/ or masugi nephritis/ or membranoproliferative glomerulonephritis/ or membranous glomerulonephritis/ or minimal change glomerulonephritis/ or proliferative glomerulonephritis/ or rapidly progressive glomerulonephritis/ (71602)

6 diabetic nephropathy/ or experimental diabetic nephropathy/ or streptozotocin-induced diabetic nephropathy/ (49751)

7 chronic kidney disease*.mp. [mp=title, abstract, heading word, drug trade name, original title, device manufacturer, drug manufacturer, device trade name, keyword heading word, floating subheading word, candidate term word] (119278)

8 CKD.mp. [mp=title, abstract, heading word, drug trade name, original title, device manufacturer, drug manufacturer, device trade name, keyword heading word, floating subheading word, candidate term word] (69868)

9 chronic kidney failure.mp. [mp=title, abstract, heading word, drug trade name, original title, device manufacturer, drug manufacturer, device trade name, keyword heading word, floating subheading word, candidate term word] (127722)

10 chronic kidney disorder*.mp. [mp=title, abstract, heading word, drug trade name, original title, device manufacturer, drug manufacturer, device trade name, keyword heading word, floating subheading word, candidate term word] (69)

11 chronic kidney insufficienc*.mp. [mp=title, abstract, heading word, drug trade name, original title, device manufacturer, drug manufacturer, device trade name, keyword heading word, floating subheading word, candidate term word] (253)

12 chronic kidney dysfunction*.mp. [mp=title, abstract, heading word, drug trade name, original title, device manufacturer, drug manufacturer, device trade name, keyword heading word, floating subheading word, candidate term word] (134)

13 chronic renal disease*.mp. [mp=title, abstract, heading word, drug trade name, original title, device manufacturer, drug manufacturer, device trade name, keyword heading word, floating subheading word, candidate term word] (6945)

14 chronic renal failure.mp. [mp=title, abstract, heading word, drug trade name, original title, device manufacturer, drug manufacturer, device trade name, keyword heading word, floating subheading word, candidate term word] (35629)

15 chronic renal insufficienc*.mp. [mp=title, abstract, heading word, drug trade name, original title, device manufacturer, drug manufacturer, device trade name, keyword heading word, floating subheading word, candidate term word] (7627)

16 chronic renal disorder*.mp. [mp=title, abstract, heading word, drug trade name, original title, device manufacturer, drug manufacturer, device trade name, keyword heading word, floating subheading word, candidate term word] (87)

17 chronic renal dysfunction*.mp. [mp=title, abstract, heading word, drug trade name, original title, device manufacturer, drug manufacturer, device trade name, keyword heading word, floating subheading word, candidate term word] (507)

18 nephropath*.mp. [mp=title, abstract, heading word, drug trade name, original title, device manufacturer, drug manufacturer, device trade name, keyword heading word, floating subheading word, candidate term word] (123221)

19 nephritis.mp. [mp=title, abstract, heading word, drug trade name, original title, device manufacturer, drug manufacturer, device trade name, keyword heading word, floating subheading word, candidate term word] (65692)

20 End stage kidney disease*.mp. [mp=title, abstract, heading word, drug trade name, original title, device manufacturer, drug manufacturer, device trade name, keyword heading word, floating subheading word, candidate term word] (8151)

21 endstage kidney disease*.mp. [mp=title, abstract, heading word, drug trade name, original title, device manufacturer, drug manufacturer, device trade name, keyword heading word, floating subheading word, candidate term word] (138)

22 End stage kidney failure.mp. [mp=title, abstract, heading word, drug trade name, original title, device manufacturer, drug manufacturer, device trade name, keyword heading word, floating subheading word, candidate term word] (426)

23 End stage renal failure.mp. [mp=title, abstract, heading word, drug trade name, original title, device manufacturer, drug manufacturer, device trade name, keyword heading word, floating subheading word, candidate term word] (9221)

24 Endstage renal failure.mp. [mp=title, abstract, heading word, drug trade name, original title, device manufacturer, drug manufacturer, device trade name, keyword heading word, floating subheading word, candidate term word] (305)

25 End stage renal insufficienc*.mp. [mp=title, abstract, heading word, drug trade name, original title, device manufacturer, drug manufacturer, device trade name, keyword heading word, floating subheading word, candidate term word] (104)

26 end stage renal disease*.mp. [mp=title, abstract, heading word, drug trade name, original title, device manufacturer, drug manufacturer, device trade name, keyword heading word, floating subheading word, candidate term word] (78278)

27 endstage renal disease*.mp. [mp=title, abstract, heading word, drug trade name, original title, device manufacturer, drug manufacturer, device trade name, keyword heading word, floating subheading word, candidate term word] (1230)

28 ERSD.mp. [mp=title, abstract, heading word, drug trade name, original title, device manufacturer, drug manufacturer, device trade name, keyword heading word, floating subheading word, candidate term word] (172)

29 ESKD.mp. [mp=title, abstract, heading word, drug trade name, original title, device manufacturer, drug manufacturer, device trade name, keyword heading word, floating subheading word, candidate term word] (3596)

30 end stage renal dysfunction*.mp. [mp=title, abstract, heading word, drug trade name, original title, device manufacturer, drug manufacturer, device trade name, keyword heading word, floating subheading word, candidate term word] (49)

31 end stage renal disorder*.mp. [mp=title, abstract, heading word, drug trade name, original title, device manufacturer, drug manufacturer, device trade name, keyword heading word, floating subheading word, candidate term word] (20)

32 Hemodialysis.mp. [mp=title, abstract, heading word, drug trade name, original title, device manufacturer, drug manufacturer, device trade name, keyword heading word, floating subheading word, candidate term word] (172865)

33 Haemodialysis.mp. [mp=title, abstract, heading word, drug trade name, original title, device manufacturer, drug manufacturer, device trade name, keyword heading word, floating subheading word, candidate term word] (23963)

34 blood dialysis.mp. [mp=title, abstract, heading word, drug trade name, original title, device manufacturer, drug manufacturer, device trade name, keyword heading word, floating subheading word, candidate term word] (90)

35 peritoneal dialysis.mp. [mp=title, abstract, heading word, drug trade name, original title, device manufacturer, drug manufacturer, device trade name, keyword heading word, floating subheading word, candidate term word] (53649)

36 peritoneum dialysis.mp. [mp=title, abstract, heading word, drug trade name, original title, device manufacturer, drug manufacturer, device trade name, keyword heading word, floating subheading word, candidate term word] (14)

37 kidney transplant*.mp. [mp=title, abstract, heading word, drug trade name, original title, device manufacturer, drug manufacturer, device trade name, keyword heading word, floating subheading word, candidate term word] (164392)

38 kidney graft*.mp. [mp=title, abstract, heading word, drug trade name, original title, device manufacturer, drug manufacturer, device trade name, keyword heading word, floating subheading word, candidate term word] (67273)

39 renal transplant*.mp. [mp=title, abstract, heading word, drug trade name, original title, device manufacturer, drug manufacturer, device trade name, keyword heading word, floating subheading word, candidate term word] (74984)

40 renal graft*.mp. [mp=title, abstract, heading word, drug trade name, original title, device manufacturer, drug manufacturer, device trade name, keyword heading word, floating subheading word, candidate term word] (7283)

41 glomerulonephritis.mp. [mp=title, abstract, heading word, drug trade name, original title, device manufacturer, drug manufacturer, device trade name, keyword heading word, floating subheading word, candidate term word] (70854)

42 diabetic kidney disease*.mp. [mp=title, abstract, heading word, drug trade name, original title, device manufacturer, drug manufacturer, device trade name, keyword heading word, floating subheading word, candidate term word] (5622)

43 diabetic glomerulopath*.mp. [mp=title, abstract, heading word, drug trade name, original title, device manufacturer, drug manufacturer, device trade name, keyword heading word, floating subheading word, candidate term word] (462)

44 diabetic glomerulosclerosis.mp. [mp=title, abstract, heading word, drug trade name, original title, device manufacturer, drug manufacturer, device trade name, keyword heading word, floating subheading word, candidate term word] (809)

45 or/1-44 (717694)

46 vitamin d/ or 24,25 dihydroxyvitamin d/ or 25 hydroxyvitamin d/ or "9,10 secocholesta 5,7,10(19) trien 23 yne 1,3,25 triol"/ or "9,10 secocholesta 5,7,10(19) trien 23 yne 3,25 diol"/ or "9,10 secocholesta 5,7,10(19),16 tetraen 23 yne 1,3,25 triol"/ or "9,10 secocholesta 5,7,10(19),22 tetraene 1,3,25,26 tetrol"/ or ascorbic acid plus fluoride plus retinol plus vitamin d/ or calcium carbonate plus ferrous fumarate plus vitamin d/ or calcium phosphate dibasic plus ferrous sulfate plus manganese sulfate plus nicotinic acid plus riboflavin plus thiamine plus vitamin d/ or dihydrotachysterol/ or lunacalcipol/ or vitamin d derivative/ (111364)

47 ergocalciferol derivative/ or 1,25 dihydroxy 24 epiergocalciferol/ or 1,25 dihydroxyergocalciferol/ or 1,25,26 trihydroxyergocalciferol/ or 1,25,28 trihydroxyergocalciferol/ or 22,23 dihydroergocalciferol/ or 24,25 dihydroxyergocalciferol/ or 25 hydroxyergocalciferol/ or 25,28 dihydroxyergocalciferol/ or doxercalciferol/ or ergocalciferol/ or paricalcitol/ (14105)

48 colecalciferol derivative/ or 1,23,25 trihydroxycolecalciferol/ or 1,24,25 trihydroxycolecalciferol/ or 1beta,25 dihydroxycolecalciferol/ or "20 epi 22 ethoxy 24a,26a,27a trihomo 9,10 secocholesta 5,7,10(19) trien 23 yne 1alpha,3beta,25 triol"/ or 25,26 dihydroxycolecalciferol/ or alendronic acid plus colecalciferol/ or atocalcitol/ or becocalcidiol/ or betamethasone dipropionate plus calcipotriol/ or calcifediol/ or calcipotriol/ or calcium carbonate plus colecalciferol plus ibandronic acid/ or calcium carbonate plus colecalciferol plus risedronic acid/ or calcium carbonate plus colecalciferol plus zoledronic acid/ or calcium plus colecalciferol/ or colecalciferol/ or colecalciferol plus strontium ranelate/ or dihydroxycolecalciferol/ or ecalcidene/ or eldecalcitol/ or elocalcitol/ or hydroxycolecalciferol/ or inecalcitol/ or lexacalcitol/ or pefcalcitol/ or secalciferol/ or seocalcitol/ or tisocalcitate/ (40983)

49 vitamin d.mp. [mp=title, abstract, heading word, drug trade name, original title, device manufacturer, drug manufacturer, device trade name, keyword heading word, floating subheading word, candidate term word] (148373)

50 cholecalciferol.mp. [mp=title, abstract, heading word, drug trade name, original title, device manufacturer, drug manufacturer, device trade name, keyword heading word, floating subheading word, candidate term word] (5104)

51 colecalciferol.mp. [mp=title, abstract, heading word, drug trade name, original title, device manufacturer, drug manufacturer, device trade name, keyword heading word, floating subheading word, candidate term word] (27882)

52 ergocalciferol.mp. [mp=title, abstract, heading word, drug trade name, original title, device manufacturer, drug manufacturer, device trade name, keyword heading word, floating subheading word, candidate term word] (10978)

53 calcifediol.mp. [mp=title, abstract, heading word, drug trade name, original title, device manufacturer, drug manufacturer, device trade name, keyword heading word, floating subheading word, candidate term word] (10528)

54 or/46-53 (176509)

55 Randomized controlled trial/ (709180)

56 Controlled clinical study/ (465872)

57 random$.ti,ab. (1794466)

58 randomization/ (93988)

59 intermethod comparison/ (282516)

60 placebo.ti,ab. (344862)

61 (compare or compared or comparison).ti. (594149)

62 ((evaluated or evaluate or evaluating or assessed or assess) and (compare or compared or comparing or comparison)).ab. (2494576)

63 (open adj label).ti,ab. (96515)

64 ((double or single or doubly or singly) adj (blind or blinded or blindly)).ti,ab. (261329)

65 double blind procedure/ (196987)

66 parallel group$1.ti,ab. (29307)

67 (crossover or cross over).ti,ab. (117328)

68 ((assign$ or match or matched or allocation) adj5 (alternate or group$1 or intervention$1 or patient$1 or subject$1 or participant$1)).ti,ab. (380433)

69 (assigned or allocated).ti,ab. (448259)

70 (controlled adj7 (study or design or trial)).ti,ab. (409679)

71 (volunteer or volunteers).ti,ab.(273285)

72 human experiment/ (573910)

73 trial.ti. (365254)

74 or/55-73 (5822074)

75 (random$ adj sampl$ adj7 (cross section$ or questionnaire$1 or survey$ or database$1)).ti,ab. not (comparative study/ or controlled study/ or randomi?ed controlled.ti,ab. or randomly assigned.ti,ab.) (9056)

76 Cross-sectional study/ not (randomized controlled trial/ or controlled clinical study/ or controlled study/ or randomi?ed controlled.ti,ab. or control group$1.ti,ab.) (307449)

77 (((case adj control$) and random$) not randomi?ed controlled).ti,ab. (19748)

78 (Systematic review not (trial or study)).ti. (207844)

79 (nonrandom$ not random$).ti,ab. (17863)

80 "Random field$".ti,ab. (2693)

81 (random cluster adj3 sampl$).ti,ab. (1433)

82 (review.ab. and review.pt.) not trial.ti. (988850)

83 "we searched".ab. and (review.ti. or review.pt.) (41601)

84 "update review".ab. (122)

85 (databases adj4 searched).ab. (50313)

86 (rat or rats or mouse or mice or swine or porcine or murine or sheep or lambs or pigs or piglets or rabbit or rabbits or cat or cats or dog or dogs or cattle or bovine or monkey or monkeys or trout or marmoset$1).ti. and animal experiment/ (1147757)

87 Animal experiment/ not (human experiment/ or human/) (2412365)

88 or/75-87 (3952717)

89 74 not 88 (5166159)

90 45 and 54 and 89 (2935)

91 90 not ((exp infant/ or exp child/ or adolescent/) not exp adult/) (2786)

**Ovid MEDLINE(R) ALL <1946 to May 02, 2022>**

1 renal insufficiency, chronic/ or kidney failure, chronic/ or frasier syndrome/ or "chronic kidney disease-mineral and bone disorder"/ (129960)

2 renal dialysis/ or hemodiafiltration/ or hemodialysis, home/ or peritoneal dialysis/ or peritoneal dialysis, continuous ambulatory/ (122014)

3 Kidney Transplantation/ (102302)

4 glomerulonephritis/ or anti-glomerular basement membrane disease/ or glomerulonephritis, iga/ or glomerulonephritis, membranoproliferative/ or glomerulonephritis, membranous/ or glomerulosclerosis, focal segmental/ or lupus nephritis/ (50352)

5 Diabetic Nephropathies/ (28066)

6 chronic kidney disease*.mp. [mp=title, abstract, original title, name of substance word, subject heading word, floating sub-heading word, keyword heading word, organism supplementary concept word, protocol supplementary concept word, rare disease supplementary concept word, unique identifier, synonyms] (67154)

7 CKD.mp. [mp=title, abstract, original title, name of substance word, subject heading word, floating sub-heading word, keyword heading word, organism supplementary concept word, protocol supplementary concept word, rare disease supplementary concept word, unique identifier, synonyms] (37708)

8 chronic kidney failure.mp. [mp=title, abstract, original title, name of substance word, subject heading word, floating sub-heading word, keyword heading word, organism supplementary concept word, protocol supplementary concept word, rare disease supplementary concept word, unique identifier, synonyms] (1576)

9 chronic kidney disorder*.mp. [mp=title, abstract, original title, name of substance word, subject heading word, floating sub-heading word, keyword heading word, organism supplementary concept word, protocol supplementary concept word, rare disease supplementary concept word, unique identifier, synonyms] (48)

10 chronic kidney insufficienc*.mp. [mp=title, abstract, original title, name of substance word, subject heading word, floating sub-heading word, keyword heading word, organism supplementary concept word, protocol supplementary concept word, rare disease supplementary concept word, unique identifier, synonyms] (239)

11 chronic kidney dysfunction*.mp. [mp=title, abstract, original title, name of substance word, subject heading word, floating sub-heading word, keyword heading word, organism supplementary concept word, protocol supplementary concept word, rare disease supplementary concept word, unique identifier, synonyms] (80)

12 chronic renal disease*.mp. [mp=title, abstract, original title, name of substance word, subject heading word, floating sub-heading word, keyword heading word, organism supplementary concept word, protocol supplementary concept word, rare disease supplementary concept word, unique identifier, synonyms] (4125)

13 chronic renal failure.mp. [mp=title, abstract, original title, name of substance word, subject heading word, floating sub-heading word, keyword heading word, organism supplementary concept word, protocol supplementary concept word, rare disease supplementary concept word, unique identifier, synonyms] (24429)

14 chronic renal insufficienc*.mp. [mp=title, abstract, original title, name of substance word, subject heading word, floating sub-heading word, keyword heading word, organism supplementary concept word, protocol supplementary concept word, rare disease supplementary concept word, unique identifier, synonyms] (5305)

15 chronic renal disorder*.mp. [mp=title, abstract, original title, name of substance word, subject heading word, floating sub-heading word, keyword heading word, organism supplementary concept word, protocol supplementary concept word, rare disease supplementary concept word, unique identifier, synonyms] (54)

16 chronic renal dysfunction*.mp. [mp=title, abstract, original title, name of substance word, subject heading word, floating sub-heading word, keyword heading word, organism supplementary concept word, protocol supplementary concept word, rare disease supplementary concept word, unique identifier, synonyms] (279)

17 nephropath*.mp. [mp=title, abstract, original title, name of substance word, subject heading word, floating sub-heading word, keyword heading word, organism supplementary concept word, protocol supplementary concept word, rare disease supplementary concept word, unique identifier, synonyms] (77135)

18 nephritis.mp. [mp=title, abstract, original title, name of substance word, subject heading word, floating sub-heading word, keyword heading word, organism supplementary concept word, protocol supplementary concept word, rare disease supplementary concept word, unique identifier, synonyms] (36071)

19 End stage kidney disease*.mp. [mp=title, abstract, original title, name of substance word, subject heading word, floating sub-heading word, keyword heading word, organism supplementary concept word, protocol supplementary concept word, rare disease supplementary concept word, unique identifier, synonyms] (5300)

20 endstage kidney disease*.mp. [mp=title, abstract, original title, name of substance word, subject heading word, floating sub-heading word, keyword heading word, organism supplementary concept word, protocol supplementary concept word, rare disease supplementary concept word, unique identifier, synonyms] (27)

21 End stage kidney failure.mp. [mp=title, abstract, original title, name of substance word, subject heading word, floating sub-heading word, keyword heading word, organism supplementary concept word, protocol supplementary concept word, rare disease supplementary concept word, unique identifier, synonyms] (272)

22 End stage renal failure.mp. [mp=title, abstract, original title, name of substance word, subject heading word, floating sub-heading word, keyword heading word, organism supplementary concept word, protocol supplementary concept word, rare disease supplementary concept word, unique identifier, synonyms] (6410)

23 Endstage renal failure.mp. [mp=title, abstract, original title, name of substance word, subject heading word, floating sub-heading word, keyword heading word, organism supplementary concept word, protocol supplementary concept word, rare disease supplementary concept word, unique identifier, synonyms] (147)

24 End stage renal insufficienc*.mp. [mp=title, abstract, original title, name of substance word, subject heading word, floating sub-heading word, keyword heading word, organism supplementary concept word, protocol supplementary concept word, rare disease supplementary concept word, unique identifier, synonyms] (77)

25 end stage renal disease*.mp. [mp=title, abstract, original title, name of substance word, subject heading word, floating sub-heading word, keyword heading word, organism supplementary concept word, protocol supplementary concept word, rare disease supplementary concept word, unique identifier, synonyms] (37041)

26 endstage renal disease*.mp. [mp=title, abstract, original title, name of substance word, subject heading word, floating sub-heading word, keyword heading word, organism supplementary concept word, protocol supplementary concept word, rare disease supplementary concept word, unique identifier, synonyms] (379)

27 ERSD.mp. [mp=title, abstract, original title, name of substance word, subject heading word, floating sub-heading word, keyword heading word, organism supplementary concept word, protocol supplementary concept word, rare disease supplementary concept word, unique identifier, synonyms] (78)

28 ESKD.mp. [mp=title, abstract, original title, name of substance word, subject heading word, floating sub-heading word, keyword heading word, organism supplementary concept word, protocol supplementary concept word, rare disease supplementary concept word, unique identifier, synonyms] (2037)

29 end stage renal dysfunction*.mp. [mp=title, abstract, original title, name of substance word, subject heading word, floating sub-heading word, keyword heading word, organism supplementary concept word, protocol supplementary concept word, rare disease supplementary concept word, unique identifier, synonyms] (28)

30 end stage renal disorder*.mp. [mp=title, abstract, original title, name of substance word, subject heading word, floating sub-heading word, keyword heading word, organism supplementary concept word, protocol supplementary concept word, rare disease supplementary concept word, unique identifier, synonyms] (11)

31 Hemodialysis.mp. [mp=title, abstract, original title, name of substance word, subject heading word, floating sub-heading word, keyword heading word, organism supplementary concept word, protocol supplementary concept word, rare disease supplementary concept word, unique identifier, synonyms] (71722)

32 Haemodialysis.mp. [mp=title, abstract, original title, name of substance word, subject heading word, floating sub-heading word, keyword heading word, organism supplementary concept word, protocol supplementary concept word, rare disease supplementary concept word, unique identifier, synonyms] (16153)

33 blood dialysis.mp. [mp=title, abstract, original title, name of substance word, subject heading word, floating sub-heading word, keyword heading word, organism supplementary concept word, protocol supplementary concept word, rare disease supplementary concept word, unique identifier, synonyms] (56)

34 peritoneal dialysis.mp. [mp=title, abstract, original title, name of substance word, subject heading word, floating sub-heading word, keyword heading word, organism supplementary concept word, protocol supplementary concept word, rare disease supplementary concept word, unique identifier, synonyms] (34493)

35 kidney transplant*.mp. [mp=title, abstract, original title, name of substance word, subject heading word, floating sub-heading word, keyword heading word, organism supplementary concept word, protocol supplementary concept word, rare disease supplementary concept word, unique identifier, synonyms] (112654)

36 kidney graft*.mp. [mp=title, abstract, original title, name of substance word, subject heading word, floating sub-heading word, keyword heading word, organism supplementary concept word, protocol supplementary concept word, rare disease supplementary concept word, unique identifier, synonyms] (4299)

37 renal transplant*.mp. [mp=title, abstract, original title, name of substance word, subject heading word, floating sub-heading word, keyword heading word, organism supplementary concept word, protocol supplementary concept word, rare disease supplementary concept word, unique identifier, synonyms] (48393)

38 renal graft*.mp. [mp=title, abstract, original title, name of substance word, subject heading word, floating sub-heading word, keyword heading word, organism supplementary concept word, protocol supplementary concept word, rare disease supplementary concept word, unique identifier, synonyms] (3314)

39 glomerulonephritis.mp. [mp=title, abstract, original title, name of substance word, subject heading word, floating sub-heading word, keyword heading word, organism supplementary concept word, protocol supplementary concept word, rare disease supplementary concept word, unique identifier, synonyms] (48450)

40 diabetic kidney disease*.mp. [mp=title, abstract, original title, name of substance word, subject heading word, floating sub-heading word, keyword heading word, organism supplementary concept word, protocol supplementary concept word, rare disease supplementary concept word, unique identifier, synonyms] (3854)

41 diabetic glomerulopath*.mp. [mp=title, abstract, original title, name of substance word, subject heading word, floating sub-heading word, keyword heading word, organism supplementary concept word, protocol supplementary concept word, rare disease supplementary concept word, unique identifier, synonyms] (343)

42 diabetic glomerulosclerosis.mp. [mp=title, abstract, original title, name of substance word, subject heading word, floating sub-heading word, keyword heading word, organism supplementary concept word, protocol supplementary concept word, rare disease supplementary concept word, unique identifier, synonyms] (555)

43 or/1-42 (466945)

44 vitamin d/ or cholecalciferol/ or hydroxycholecalciferols/ or calcifediol/ or dihydroxycholecalciferols/ or 24,25-dihydroxyvitamin d 3/ or ergocalciferols/ or dihydrotachysterol/ or 25-hydroxyvitamin d 2/ (54687)

45 vitamin d.mp. [mp=title, abstract, original title, name of substance word, subject heading word, floating sub-heading word, keyword heading word, organism supplementary concept word, protocol supplementary concept word, rare disease supplementary concept word, unique identifier, synonyms] (81191)

46 cholecalciferol.mp. [mp=title, abstract, original title, name of substance word, subject heading word, floating sub-heading word, keyword heading word, organism supplementary concept word, protocol supplementary concept word, rare disease supplementary concept word, unique identifier, synonyms] (9668)

47 colecalciferol.mp. [mp=title, abstract, original title, name of substance word, subject heading word, floating sub-heading word, keyword heading word, organism supplementary concept word, protocol supplementary concept word, rare disease supplementary concept word, unique identifier, synonyms] (82)

48 ergocalciferol.mp. [mp=title, abstract, original title, name of substance word, subject heading word, floating sub-heading word, keyword heading word, organism supplementary concept word, protocol supplementary concept word, rare disease supplementary concept word, unique identifier, synonyms] (803)

49 calcifediol.mp. [mp=title, abstract, original title, name of substance word, subject heading word, floating sub-heading word, keyword heading word, organism supplementary concept word, protocol supplementary concept word, rare disease supplementary concept word, unique identifier, synonyms] (4430)

50 or/44-49 (89029)

51 randomized controlled trial.pt. (566697)

52 controlled clinical trial.pt. (94847)

53 random*.ab. (1273269)

54 placebo.ab. (227712)

55 drug therapy.fs (2482838)

56 trial.ab.(597367)

57 groups.ab. (2342735)

58 or/51-57 (5582292)

59 exp animals/ not humans.sh. (4999748)

60 58 not 59 (4864572)

61 43 and 50 and 60 (3116)

62 61 not ((exp infant/ or exp child/ or adolescent/) not exp adult/) (2884)

63 review.pt. not trial.ti.

64 62 not 63 (2204)

**EBM Reviews - Cochrane Central Register of Controlled Trials <March 2022>**

1 renal insufficiency, chronic/ or kidney failure, chronic/ or frasier syndrome/ or "chronic kidney disease-mineral and bone disorder"/ (7406)

2 renal dialysis/ or hemodiafiltration/ or hemodialysis, home/ or peritoneal dialysis/ or peritoneal dialysis, continuous ambulatory/ (5482)

3 Kidney Transplantation/ (3705)

4 glomerulonephritis/ or anti-glomerular basement membrane disease/ or glomerulonephritis, iga/ or glomerulonephritis, membranoproliferative/ or glomerulonephritis, membranous/ or glomerulosclerosis, focal segmental/ or lupus nephritis/ (963)

5 Diabetic Nephropathies/ (1526)

6 chronic kidney disease*.mp. [mp=title, original title, abstract, floating sub-heading word, mesh headings, heading words, keyword] (9193)

7 CKD.mp. [mp=title, original title, abstract, floating sub-heading word, mesh headings, heading words, keyword] (6724)

8 chronic kidney failure.mp. [mp=title, original title, abstract, floating sub-heading word, mesh headings, heading words, keyword] (4061)

9 chronic kidney disorder*.mp. [mp=title, original title, abstract, floating sub-heading word, mesh headings, heading words, keyword] (4)

10 chronic kidney insufficienc*.mp. [mp=title, original title, abstract, floating sub-heading word, mesh headings, heading words, keyword] (9)

11 chronic kidney dysfunction*.mp. [mp=title, original title, abstract, floating sub-heading word, mesh headings, heading words, keyword] (15)

12 chronic renal disease*.mp. [mp=title, original title, abstract, floating sub-heading word, mesh headings, heading words, keyword] (376)

13 chronic renal failure.mp. [mp=title, original title, abstract, floating sub-heading word, mesh headings, heading words, keyword] (2159)

14 chronic renal insufficienc*.mp. [mp=title, original title, abstract, floating sub-heading word, mesh headings, heading words, keyword] (453)

15 chronic renal disorder*.mp. [mp=title, original title, abstract, floating sub-heading word, mesh headings, heading words, keyword] (1)

16 chronic renal dysfunction*.mp. [mp=title, original title, abstract, floating sub-heading word, mesh headings, heading words, keyword] (44)

17 nephropath*.mp. [mp=title, original title, abstract, floating sub-heading word, mesh headings, heading words, keyword] (8376)

18 nephritis.mp. [mp=title, original title, abstract, floating sub-heading word, mesh headings, heading words, keyword] (1535)

19 End stage kidney disease*.mp. [mp=title, original title, abstract, floating sub-heading word, mesh headings, heading words, keyword] (655)

20 endstage kidney disease*.mp. [mp=title, original title, abstract, floating sub-heading word, mesh headings, heading words, keyword] (11)

21 End stage kidney failure.mp. [mp=title, original title, abstract, floating sub-heading word, mesh headings, heading words, keyword] (30)

22 End stage renal failure.mp. [mp=title, original title, abstract, floating sub-heading word, mesh headings, heading words, keyword] (526)

23 Endstage renal failure.mp. [mp=title, original title, abstract, floating sub-heading word, mesh headings, heading words, keyword] (11)

24 End stage renal insufficienc*.mp. [mp=title, original title, abstract, floating sub-heading word, mesh headings, heading words, keyword] (4)

25 end stage renal disease*.mp. [mp=title, original title, abstract, floating sub-heading word, mesh headings, heading words, keyword] (4710)

26 endstage renal disease*.mp. [mp=title, original title, abstract, floating sub-heading word, mesh headings, heading words, keyword] (55)

27 ERSD.mp. [mp=title, original title, abstract, floating sub-heading word, mesh headings, heading words, keyword] (9)

28 ESKD.mp. [mp=title, original title, abstract, floating sub-heading word, mesh headings, heading words, keyword] (248)

29 end stage renal dysfunction*.mp. [mp=title, original title, abstract, floating sub-heading word, mesh headings, heading words, keyword] (3)

30 end stage renal disorder*.mp. [mp=title, original title, abstract, floating sub-heading word, mesh headings, heading words, keyword] (0)

31 Hemodialysis.mp. [mp=title, original title, abstract, floating sub-heading word, mesh headings, heading words, keyword] (12089)

32 Haemodialysis.mp. [mp=title, original title, abstract, floating sub-heading word, mesh headings, heading words, keyword] (2293)

33 blood dialysis.mp. [mp=title, original title, abstract, floating sub-heading word, mesh headings, heading words, keyword] (13)

34 peritoneal dialysis.mp. [mp=title, original title, abstract, floating sub-heading word, mesh headings, heading words, keyword] (2493)

35 kidney transplant*.mp. [mp=title, original title, abstract, floating sub-heading word, mesh headings, heading words, keyword] (8662)

36 kidney graft*.mp. [mp=title, original title, abstract, floating sub-heading word, mesh headings, heading words, keyword] (2638)

37 renal transplant*.mp. [mp=title, original title, abstract, floating sub-heading word, mesh headings, heading words, keyword] (5881)

38 renal graft*.mp. [mp=title, original title, abstract, floating sub-heading word, mesh headings, heading words, keyword] (305)

39 glomerulonephritis.mp. [mp=title, original title, abstract, floating sub-heading word, mesh headings, heading words, keyword] (1512)

40 diabetic kidney disease*.mp. [mp=title, original title, abstract, floating sub-heading word, mesh headings, heading words, keyword] (508)

41 diabetic glomerulopath*.mp. [mp=title, original title, abstract, floating sub-heading word, mesh headings, heading words, keyword] (21)

42 diabetic glomerulosclerosis.mp. [mp=title, original title, abstract, floating sub-heading word, mesh headings, heading words, keyword] (7)

43 or/1-42 (46124)

44 vitamin d/ or cholecalciferol/ or hydroxycholecalciferols/ or calcifediol/ or dihydroxycholecalciferols/ or 24,25-dihydroxyvitamin d 3/ or ergocalciferols/ or dihydrotachysterol/ or 25-hydroxyvitamin d 2/ (5300)

45 vitamin d.mp. [mp=title, original title, abstract, floating sub-heading word, mesh headings, heading words, keyword] (13641)

46 cholecalciferol.mp. [mp=title, original title, abstract, floating sub-heading word, mesh headings, heading words, keyword] (3264)

47 colecalciferol.mp. [mp=title, original title, abstract, floating sub-heading word, mesh headings, heading words, keyword] (978)

48 ergocalciferol.mp. [mp=title, original title, abstract, floating sub-heading word, mesh headings, heading words, keyword] (328)

49 calcifediol.mp. [mp=title, original title, abstract, floating sub-heading word, mesh headings, heading words, keyword] (610)

50 or/44-49 (14411)

51 43 and 50 (1347)

52 51 not ((exp infant/ or exp child/ or adolescent/) not exp adult/) (1321)

**Web of Science**

**#1** TS=(volunteer or volunteers)

**#2** TS=((controlled) NEAR/7 (study or design or trial))

**#3** TS=(assigned or allocated)

**#4** TS=((assign or assigns or match or matched or allocation) NEAR/5 (alternate or group or groups or intervention or interventions or patient or patients or subject or subjects or participant or participants))

**#5** TS=((double or single or doubly or singly) NEAR (blind or blinded or blindly))

**#6** TS=(open NEAR label)

**#7** AB=((evaluated or evaluate or evaluating or assessed or assess) and (compare or compared or comparing or comparison))

**#8** TI=(compare or compared or comparison)

**#9** AB=groups

**#10** ((TS=("controlled clinical" OR "control group" OR trial or placebo OR "drug therap*" OR random* OR "parallel group" OR "parallel groups" OR crossover OR "cross over")))

**#11** #10 OR #9 OR #8 OR #7 OR #6 OR #5 OR #4 OR #3 OR #2 OR #1

**#12** ((TS=("chronic kidney disease*" OR CKD OR "chronic kidney failure" OR "chronic kidney disorder*" OR "chronic kidney insufficienc*" OR "chronic kidney dysfunction*" OR "chronic renal disease*" OR "chronic renal failure" OR "chronic renal insufficienc*" OR "chronic renal disorder*" OR "chronic renal dysfunction*" OR nephropath* OR nephritis OR "End stage kidney disease*" OR "endstage kidney disease*" OR "End stage kidney failure" OR "End stage renal failure" OR "Endstage renal failure" OR "End stage renal insufficienc*" OR "end stage renal disease*" OR "endstage renal disease*" OR ERSD OR EKSD OR "end stage renal dysfunction*" OR "end stage renal disorder*" OR Hemodialysis OR Haemodialysis OR "blood dialysis" OR "peritoneal dialysis" OR "kidney transplant*" OR "kidney graft*" OR "renal transplant*" OR "renal graft*" OR glomerulonephritis OR "diabetic kidney disease*" OR "diabetic glomerulopath*" OR "diabetic glomerulosclerosis")))

**#13** TS=("vitamin d" OR cholecalciferol OR colecalciferol OR ergocalciferol OR calcifediol OR hydroxycholecalciferol* OR dihydroxycholecalciferol* OR dihydrotachysterol)

**#14** #13 AND #12 AND #11

**#15** #13 AND #12 AND #11 and Review Articles (Exclude – Document Types) (2581)

**ProQuest Dissertations & Theses Global**

((ab("vitamin d") OR ti("vitamin d")) AND (ab(("chronic kidney disease*" OR "chronic renal disease*" OR "end stage kidney disease" OR hemodialysis OR "peritoneal dialysis" OR "kidney transplant")) OR ti(("chronic kidney disease*" OR "chronic renal disease*" OR "end stage kidney disease" OR hemodialysis OR "peritoneal dialysis" OR "kidney transplant")))) AND (ab(("chronic kidney disease*" OR "chronic renal disease*" OR "end stage kidney disease" OR hemodialysis OR "peritoneal dialysis" OR "kidney transplant")) OR ti(("chronic kidney disease*" OR "chronic renal disease*" OR "end stage kidney disease" OR hemodialysis OR "peritoneal dialysis" OR "kidney transplant")))

82 total results

**medRxiv**

*Searched separately in title and abstract field:*

Vitamin d

cholecalciferol

colecalciferol

ergocalciferol

calcifediol

hydroxycholecalciferol

dihydroxycholecalciferol

dihydrotachysterol

151 total results
